# Laparoscopic versus open distal pancreatectomy: a single centre propensity score matching analysis

**DOI:** 10.1007/s13304-021-01039-x

**Published:** 2021-04-03

**Authors:** Riccardo Casadei, Carlo Ingaldi, Claudio Ricci, Laura Alberici, Emilio De Raffele, Maria Chiara Vaccaro, Francesco Minni

**Affiliations:** 1 Division of Pancreatic Surgery, IRCCS, Azienda Ospedaliero Universitaria di Bologna, Bologna, Italy; 2 Department of Internal Medicine and Surgery (DIMEC), Alma Mater Studiorum, University of Bologna, S. Orsola-Malpighi Hospital, Via Massarenti n.9, 40138 Bologna, Italy

**Keywords:** Laparoscopic, Open, Distal pancreatectomy, Propensity score matching, Pancreas

## Abstract

The laparoscopic approach is considered as standard practice in patients with body-tail pancreatic neoplasms. However, only a few randomized controlled trials (RCTs) and propensity score matching (PSM) studies have been performed. Thus, additional studies are needed to obtain more robust evidence. This is a single-centre propensity score-matched study including patients who underwent laparoscopic (LDP) and open distal pancreatectomy (ODP) with splenectomy for pancreatic neoplasms. Demographic, intra, postoperative and oncological data were collected. The primary endpoint was the length of hospital stay. The secondary endpoints included the assessment of the operative findings, postoperative outcomes, oncological outcomes (only in the subset of patients with pancreatic ductal adenocarcinoma-PDAC) and total costs. In total, 205 patients were analysed: 105 (51.2%) undergoing an open approach and 100 (48.8%) a laparoscopic approach. After PSM, two well-balanced groups of 75 patients were analysed and showed a shorter length of hospital stay (*P* = 0.001), a lower blood loss (*P* = 0.032), a reduced rate of postoperative morbidity (*P* < 0.001) and decreased total costs (*P* = 0.050) after LDP with respect to ODP. Regarding the subset of patients with PDAC, 22 patients were analysed: they showed a significant shorter length of hospital stay (*P* = 0.050) and a reduction in postoperative morbidity (*P* < 0.001) after LDP with respect to ODP. Oncological outcomes were similar. LDP showed lower hospital stay and postoperative morbidity rate than ODP both in the entire population and in patients affected by PDAC. Total costs were reduced only in the entire population. Oncological outcomes were comparable in PDAC patients.

## Introduction

In recent years, many systematic reviews and meta-analyses have demonstrated the feasibility and safety of laparoscopic distal pancreatectomy (LDP) for both benign and malignant pancreatic lesions, reporting postoperative outcomes at least comparable to those obtained with the open approach [1–7]. However, randomised controlled trials (RCTs) were lacking. Thus, the results were of low quality, with a high risk of bias because all the studies included in the systematic reviews and meta-analyses were retrospective or observational. In the last years, an increase in the level of evidence has occurred and two RCTs [8, 9] and five PSM studies [10–14] have been carried out. However, the two RCTs involved a small sample of patients, and the four out of five studies in which a PSM analysis was used, regarded only patients with pancreatic neuroendocrine tumours (pNETs) [14] or PDAC [11–13]. Therefore, additional comparisons of laparoscopic and open distal pancreatectomy, using reliable methods, are needed to obtain more robust evidence and to consider LDP as standard practice in patients with both benign and malignant body-tail pancreatic lesions. In this context, the present single tertiary centre study aimed at demonstrating that, using PSM to minimise the selection biases by equating the groups compared, the laparoscopic approach was not inferior to the open procedure, in the entire patient population with pancreatic neoplasms and in those patients with PDAC, regarding operative findings, postoperative outcomes, oncological outcomes and total costs.

## Methods

### Study design

This was a retrospective study based on a prospectively maintained database of patients who underwent consecutive distal pancreatectomy (DP) with splenectomy from January 2004 to January 2020. The study was approved by the Ethical Committee of S. Orsola-Malpighi Hospital (642017 U/Oss), and patient informed consent was obtained from all the participants included in the study. The patients who underwent DP were divided into two groups and were compared according to the type of approach: laparoscopic distal pancreatectomy (LDP) and open distal pancreatectomy (ODP). The following baseline characteristics were collected for each patient: gender, age, co-morbidities, American Society of Anesthesiologists (ASA) score, Body Mass Index (BMI), previous abdominal surgery, tumour size and site, and malignancy. The operative and postoperative data [operative time, blood loss, 90-day mortality, postoperative morbidity, severe postoperative morbidity, clinically relevant postoperative pancreatic fistula (CR-POPF), postoperative pancreatic fistula grade C, post-pancreatectomy haemorrhage (PHH), delayed gastric emptying (DGE), reoperation rate, readmission rate, length of hospital stay and total cost of the procedures] were also reported. Finally, a subset of patients with PDAC was analysed, also considering oncological outcomes (R0 resection, lymph node harvest and lymph node ratio).

The primary endpoint was to evaluate the length of hospital stay. The secondary endpoints included the assessment of the operative findings (operative time, blood loss), postoperative outcomes (90-day mortality, postoperative morbidity, severe postoperative morbidity, CR-POPF, POPF grade C, PPH, DGE, reoperation, readmission), total cost of the two surgical procedures and, in a subset of patients with PDAC, also oncological outcomes (R0 resection, lymph node harvest and lymph node ratio).

### Surgical technique and postoperative course.

The same routine was applied in LDP as ODP. The surgical technique was previously reported [15]. The pancreatic transection was always performed with a stapler. For each surgeon, the learning curve for LDP was considered completed after 17 procedures, as previously reported [16], and all three surgeons who performed the LDPs (RC, CR and FM) had completed their learning curve. When the tumor was in the body of the pancreas, it meant that the tumor was located between the left border of the portal vein and the left border of aorta and a subtotal pancreatectomy have to be performed; when the tumor was located in the pancreatic tail, it meant that the tumor was distal to the left border of the aorta and a left pancreatectomy was carried out. Left pancreatectomy and subtotal pancreatectomy were defined as the transection of the pancreas on the left and on the right of the portal vein, respectively. In a subtotal pancreatectomy, the resection line was at the level of the portal vein requiring a tunneling procedure while in a left pancreatectomy, the tunneling procedure was not required. Anterior Radical Antegrade Modular PancreatoSplenectomy (RAMPS), including anterior Gerota’s fascia resection, was performed in all cases affected by PDAC starting from 2015. Posterior RAMPS, including anterior and posterior Gerota’s fascia resection and adrenalectomy, was carried out only in selected cases of PDAC. Stations 10, 11 and 18 were removed for PDAC of the pancreatic tail; 8a, 9, 10, 11 and 18 of the pancreatic body. ERAS principles were followed for the postoperative course.

### Terminology and definition

Operative time was defined as the interval from the incision to the suturing of the skin. Postoperative mortality was defined as the number of deaths occurring during hospitalisation or within 90 days after surgery. Postoperative morbidity included all complications following surgery up to the day of discharge according to the Clavien–Dindo classification [17]. Major complications were classified as Clavien–Dindo > 2 [18]. A postoperative pancreatic fistula (POPF) was defined according to the 2016 definition proposed by the International Study Group of Pancreatic Fistula (ISGPF) [19]. Postpancreatectomy haemorrhage was defined as intra-abdominal or intestinal bleeding according to the criteria of the International Study Group of Pancreatic Surgery (ISGPS) [20]. Delayed gastric emptying was defined according to the criteria of the ISGPS [21]. Reoperation was defined as any surgical procedure performed in the first 30 postoperative days or before discharge from the hospital. Length of hospital stay (LOS) was calculated as the interval from the day of surgery to the day of discharge. The total cost of the two surgical procedures was calculated in Euros and included the pre, intra and postoperative costs for the reference year 2019. The initial purchase expenses of the laparoscopic system were excluded. Postoperative costs included the hospitalisation costs, postoperative imaging studies, nutritional support, surgical reoperations or interventional postoperative procedures, the intensive care unit admission expenses and readmission costs. Overall costs were calculated by adding the intraoperative costs for each patient (operative theatre cost/hour, device costs).

### Statistical analysis

All the categorical variables were described as frequencies and percentages while the continuous variables were reported as means and standard deviation. Comparison of the two groups was carried out using Fischer’s exact test, Student’s *t* test and Pearson’s χ^2^ test. Two-tailed *P* values less than 0.05 were considered statistically significant. All statistical analyses were carried out by running the Statistical Package for the Social Science (SPSS, Chicago, IL), version 13 on a personal computer. Propensity score matching was used to minimise selection bias. The patients were matched using relevant variables with the aim of equating the complexity of the surgical cases. The relevant variables were patient-related (gender, age, co-morbidities, ASA score, BMI, previous abdominal surgery) and tumour-related (tumour size, tumour site and malignancy). Two PSM comparisons were made: LDP versus ODP in all patients and LDP versus ODP in patients with PDAC. A matched group of patients was created with a 1:1 ratio in both PSM analyses. The PSM method is closest to the neighbourhood method having a caliper width of 0.20. Standardised mean difference (SMD) was used to assess the balance of the clinical backgrounds between the two groups. An SMD < 0.2 indicated very small differences between the means (this implied that optimal balance regarding a variable was generally achieved), an SMD between 0.2 and 0.8 indicated medium differences (this implied that a fairly sufficient balance regarding a variable was generally achieved) and anSMD > 0.8 indicated considerable differences (this implied that poor balance regarding a variable was generally achieved).

## Results

From January 2004 to January 2020, a total of 205 consecutive DPs with splenectomy were performed: 105 (51.2%) using an open approach and 100 (48.8%) using a laparoscopic approach. Of the latter, 19 (19.0%) required a conversion from laparoscopic to open distal pancreatectomy while 81 (81.0%) successfully completed a laparoscopic distal pancreatectomy. The baseline characteristics of LDP versus ODP before and after PSM analysis are summarised in Table 1. Before the PSM analysis, all the patients were analysed, and several significant differences between the two groups were seen. In particular, the laparoscopic approach was preferred to ODP in younger patients (*P* = 0.036) with fewer comorbidities (*P* = 0.024) and a better ASA score (*P* = 0.007). In addition, it was preferred for small (*P* = 0.050), benign (*P* < 0.001) tumours located in the pancreatic tail (*P* = 0.001). Previous abdominal surgery represented a trend (*P* = 0.062), but it is to underline that the major part of previous operations before laparoscopic approach were appendectomy (*n* = 27), cholecystectomy (*n* = 8) and hysterectomy (*n* = 7). After PSM, two well-balanced groups of 75 patients were analysed (the SMD was almost always < 0.2, indicating an optimal balance of the two groups).

**Table 1:** Baseline characteristics before and after propensity score matching analysis of LDP versus ODP

Baseline characteristics	All patients				Propensity-matched patients			
	LDP (*n* = 100) (% or Mean, SD)	ODP (*n* = 105) (% or Mean, SD)	*P* value	SMD	LDP (*n* = 75) (% or Mean, SD)	ODP (*n* = 75) (% or Mean, SD)	*P* value	SMD
Gender			0.399	0.148			0.512	0.148
M	41 (41.0)	50 (47.6)			32 (42.7)	37 (49.3)		
F	59 (59.0)	55 (52.4)			43 (57.3)	38 (50.7)		
Age (years)	60.4 (14.6)	64.4 (12.9)	**0.036**	0.292	61.5 (12.8)	62.8 (13.4)	0.537	0.099
BMI (Kg/m^2^)	26.1 (4.7)	25.9 (5.0)	0.818	0.041	26.0 (4.6)	25.8 (4.7)	0.762	0.043
ASA score			**0.007**	0.482			0.093	0.191
I	5 (5.0)	2 (1.9)			0 (0.0)	2 (2.7)		
II	48 (48.0)	31 (29.5)			38 (50.7)	29 (38.7)		
III	47 (47.0)	69 (65.7)			37 (49.3)	41 (54.6)		
IV	0 (0.0)	3 (2.9)			0 (0.0)	3 (4.0)		
Comorbidities			**0.024**	0.395			0.220	0.272
No	39 (39.0)	25 (23.8)			28 (37.3)	20 (26.7)		
Yes	61 (61.0)	80 (76.2)			47 (62.7)	55 (73.3)		
Previous abdominal surgery			0.062	0.317				
No	46 (46.0)	34 (32.4)			36 (48.0)	28 (37.3)	0.248	0.241
Yes	54 (54.0)	71 (67.6)			39 (52.0)	47 (62.7)		
Malignant tumours			** < 0.001**	0.750			0.725	0.103
No	78 (78.0)	50 (47.6)			53 (70.7)	50 (66.7)		
Yes	22 (22.0)	55 (52.4)			22 (29.3)	25 (33.3)		
Tumour size (mm)	32 (25)	39 (29)	**0.050**	0.275	33 (27)	38 (27)	0.239	0.192
Tumour site			**0.001**	0.629			1.000	0.045
Neck-body	64 (64.0)	89 (84.8)			60 (80.0)	59 (78.7)		
Tail	36 (36.0)	16 (15.2)			15 (20.0)	16 (21.3)		

Bold values are statistically significant. It allows to underline the important parameters

*LDP* laparoscopic distal pancreatectomy, *ODP* open distal pancreatectomy, *SMD* standardised mean difference, *BMI* Body Mass Index, *ASA* American Society of Anesthesiologists

Operative findings, postoperative outcomes and total costs before and after a propensity score matching analysis of LDP versus ODP are reported in Table 2. Before the PSM analysis, blood loss, postoperative morbidity, length of hospital stay and total costs were significantly lower in the LDP group than in the ODP group (*P* = 0.002, *P* < 0.001, *P* < 0.001 and *P* < 0.001, respectively). The PPH and readmission rates showed a trend in favour of LDP (*P* = 0.052 and 0.054, respectively). After PSM analysis, a lower blood loss (*P* = 0.032), a reduced rate of postoperative morbidity (*P* < 0.001), a shorter length of hospital stay (*P* = 0.001) and decreased total costs (*P* = 0.050) were reported after LDP with respect to ODP. The readmission rate showed a trend in favour of LDP (*P* = 0.061).

**Table 2:** Operative findings, postoperative outcomes and total costs before and after propensity score matching analysis of LDP versus ODP

Parameters	All patients				Propensity-matched patients			
	LDP (*n* = 100) (% or Mean, SD)	ODP (*n* = 105) (% or Mean,SD)	*P* value	SMD	LDP (*n* = 75) (% or Mean, SD)	ODP (*n* = 75) (% or Mean, SD)	i value	SMD
Operative time (min)	250 (76)	260 (75)	0.372	0.119	252 (73)	252 (76)	0.972	0.013
Blood loss (ml)	130 (94)	230 (305)	**0.002**	0.443	143 (104)	230 (329)	**0.032**	0.357
90-day mortality			*	*			*	*
No	100 (100)	105 (100)			75 (100)	75 (100)		
Yes	0 (0.0)	0 (0.0)			0 (0.0)	0 (0.0)		
Postoperative morbidity (C–D score)			** < 0.001**	0.668			**0.001**	0.495
No	48 (48.0)	11 (10.5)			33 (44.0)	10 (13.3)		
I	13 (13.0)	25 (23.8)			9 (12.1)	19 (25.3)		
II	28 (28.0)	56 (53.3)			25 (33.3)	37 (49.3)		
III	10 (10.0)	11 (10.5)			7 (9.3)	7 (9.3)		
IV	1 (1.0)	2 (1.9)			1 (1.3)	2 (2.8)		
Severe postoperative morbidity (C–D > 2)			0.830	0.074			1.000	0.073
No	89 (89.0)	92 (87.6)			67 (89.3)	66 (88.0)		
Yes	11 (11.0)	13 (12.4)			8 (10.7)	9 (12.0)		
CR-POPF			0.548	0.112			1.000	0.033
No	71 (71.0)	70 (66.7)			51 (68.0)	50 (66.7)		
Yes	29 (29.0)	35 (33.4)			24 (32.0)	25 (33.3)		
POPF grade C			0.237	*			0.497	*
No	98 (98.0)	105 (100.0)			75 (100)	73 (97.3)		
Yes	2 (2.0)	0 (0.0)			0 (0.0)	2 (2.7)		
PPH			**0.052**	0.447			0.265	0.334
No	90 (90.0)	84 (80.0)			66 (88.0)	60 (80.0)		
Yes	10 (10.0)	21 (20.0)			9 (12.0)	15 (20.0)		
DGE			1.000	0.101			1.000	0.131
No	96 (96.0)	100 (95.2)			71 (94.7)	70 (93.3)		
Yes	4 (4.0)	5 (4.8)			4 (5.3)	5 (6.7)		
Reoperation			0.764	0.134			1.000	< 0.001
No	94 (94.0)	100 (95.2)			71 (94.7)	71 (94.7)		
Yes	6 (6.0)	5 (4.8)			4 (5.3)	4 (5.3)		
Readmission			**0.054**	0.501			**0.061**	0.672
No	83 (83.0)	97 (92.4)			63 (84.0)	71 (94.7)		
Yes	17 (17.0)	8 (7.6)			12 (16.0)	4 (5.3)		
Length of hospital stay (days)	13 (7)	16 (9)	** < 0.001**	0.339	13 (7)	15 (7)	**0.001**	0.532
Total costs (euro)	15,896 (5603)	19,092 (7256)	** < 0.001**	0.492	16,307 (5913)	18,314 (6635)	**0.050**	0.319

Bold values are statistically significant. It allows to underline the important parameters

*LDP* laparoscopic distal pancreatectomy, *ODP* open distal pancreatectomy, *SMD* standardised mean difference, *C–D *Clavien–Dindo, *CR-POPF *clinically relevant-post-operative pancreatic fistula, *PPH* post-pancreatectomy haemorrhage, *DGE* delayed gastric emptying

*Not computable

Regarding the subset of patients with PDAC, the baseline characteristics before and after PSM analysis of LDP versus ODP are summarised in Table 3. Before the PSM analysis, 23 LDPs and 36 ODPs were evaluated. The only factors significantly different were age and tumour site. In particular, younger people were preferred for the laparoscopic approach (*P* = 0.039) as were those with tumours located in the pancreatic tail (*P* = 0.015). There were 22 propensity-matched patients in each group and, in the majority of them, the SMD was between 0.2 and 0.8, indicating a sufficient balance of the two groups.

**Table 3:** Baseline characteristics before and after propensity score matching analysis of LDP versus ODP in patients with pancreatic ductal adenocarcinoma

Baseline characteristics	All patients				Propensity-matched patients			
	LDP (*n* = 23) (% or Mean, SD)	ODP (*n* = 36) (% or Mean, SD)	*P* value	SMD	LDP (*n* = 22) (% or Mean, SD)	ODP (*n* = 22) (% or Mean,SD)	*P* value	SMD
Gender			0.187	0.429			1.000	< 0.001
M	9 (39.1)	21 (58.3)			9 (40.9)	9 (40.9)		
F	14 (60.9)	15 (41.7)			13 (59.1)	13 (59.1)		
Age (years)	64.7 (12.9)	70.8 (9.4)	**0.039**	0.560	65.7 (12.3)	68.9 (10.7)	0.357	0.277
BMI (Kg/m^2^)	25.6 (5.1)	24.7 (3.3)	0.486	0.196	25.8 (5.1)	24.6 (3.5)	0.388	0.251
ASA score			0.780	0.091			0.537	0.282
I	0 (0.0)	0 (0.0)			0 (0.0)	0 (0.0)		
II	8 (34.8)	11 (30.6)			7 (31.8)	10 (45.5)		
III	15 (65.2)	25 (69.4)			15 (68.2)	12 (54.5)		
IV	0 (0.0)	0 (0.0)			0 (0.0)	0 (0.0)		
Comorbidities			0.185	0.550			0.240	0.728
No	7 (30.4)	5 (13.9)			6 (27.3)	2 (9.1)		
Yes	16 (69.6)	31 (86.1)			16 (72.7)	20 (90.9)		
Previous Abdominal surgery			0.181	0.430			1.000	0.100
No	12 (52.2)	12 (33.3)			11 (50.0)	10 (45.5)		
Yes	11 (47.8)	24 (66.7)			11 (50.0)	12 (54.5)		
Tumour size (mm)	36 (25)	32 (16)	0.462	0.251	34 (24)	35 (17)	0.861	0.054
Tumour site			**0.015**	0.861			0.332	0.472
Neck-body	13 (56.5)	31 (86.1)			13 (59.1)	17 (77.3)		
Tail	10 (43.5)	5 (13.9)			9 (40.9)	5 (22.7)		

Bold values are statistically significant. It allows to underline the important parameters

*LDP* laparoscopic distal pancreatectomy, *ODP* open distal pancreatectomy, *SMD* standardised mean difference, *BMI* Body Mass Index, *ASA* American Society of Anesthesiologists

Operative findings, postoperative and oncological outcomes, and total costs before and after the PSM analysis of LDP versus ODP are reported in Table 4. Before the PSM analysis, only postoperative morbidity was significantly lower in the LDPs than in the ODPs (*P* < 0.001). Blood loss and length of hospital stay decreased, but not significantly in the LDPs with respect to the ODPs (*P* = 0.054 and 0.052, respectively). After PSM analysis, a significantly reduced rate of postoperative morbidity (*P* < 0.001) and a shorter length of hospital stay (*P* = 0.050) were confirmed after LDP with respect to ODP. The oncological outcomes (R0 resection, lymph node harvest and lymph node ratio) were comparable between the two groups.

**Table 4:** Operative findings, postoperative and oncological outcomes, and total costs before and after a propensity score matching analysis of LDP versus ODP in patients with pancreatic ductal adenocarcinoma

Parameters	All patients				Propensity-matched patients			
	**LDP (n = 23)** (% or Median, IQR)	**ODP (n = 36)** (% or Median,IQR)	***P value***	**SMD**	**LDP (n = 22)** (% or Median, IQR)	**ODP (n = 22)** (% or Median, IQR)	***P value***	**SMD**
Operative time (min)	264 (68)	270 (72)	0.733	0.086	264 (69)	262 (62)	0.906	0.137
Blood loss (ml)	123 (55)	216 (270)	0.054	0.432	125 (56.5)	238 (324)	0.112	0.486
90-day mortality			*	*			*	*
No	36 (100)	36 (100)			22 (100)	22 (100)		
Yes	0 (0.0)	0 (0.0)			0 (0.0)	0 (0.0)		
Postoperative morbidity (C–D score)			** < 0.001**	0.842			** < 0.001**	0.877
No	12 (52.2)	0 (0.0)			12 (54.4)	0 (0.0)		
I	1 (4.3)	11 (30.6)			1 (4.6)	6 (27.2)		
II	8 (34.7)	22 (61.1)			7 (31.8)	14 (63.6)		
III	1 (4.3)	2 (5.5)			1 (4.6)	1 (4.6)		
IV	1 (4.3)	1 (2.8)			1 (4.6)	1 (4.6)		
Severe postoperative morbidity (C–D > 2)			1.000	0.026			1.000	< 0.001
No	21 (91.3)	33 (91.7)			20 (90.9)	20 (90.9)		
Yes	2 (8.7)	3 (8.3)			2 (9.1)	2 (9.1)		
CR-POPF			0.578	0.209			0.747	0.232
No	14 (60.9)	25 (69.4)			14 (63.6)	16 (72.7)		
Yes	9 (39.1)	11 (30.6)			8 (36.4)	6 (27.3)		
POPF grade C			0.390	*			1.000	*
No	22 (95.7)	36 (100)			21 (95.4)	22 (100)		
Yes	1 (4.3)	0 (0.0)			1 (4.6)	0 (0.0)		
PPH			1.000	0.558			1.000	< 0.001
No	21 (91.3)	32 (88.9)			20 (90.9)	20 (90.9)		
Yes	2 (8.7)	4 (11.1)			2 (9.1)	2 (9.1)		
DGE			0.639	0.265			1.000	< 0.001
No	21 (91.3)	34 (94.4)			20 (90.9)	20 (90.9)		
Yes	2 (8.7)	2 (5.6)			2 (9.1)	2 (9.1)		
Reoperation			1.000	0.256			1.000	< 0.001
No	22 (95.6)	35 (97.2)			21 (95.4)	21 (95.4)		
Yes	1 (4.4)	1 (2.8)			1 (4.6)	1 (4.6)		
Readmission			0.369	0.516			0.607	0.661
No	20 (86.9)	34 (94.4)			19 (86.4)	21 (95.4)		
Yes	3 (13.1)	2 (5.6)			3 (13.6)	1 (4.6)		
Length of hospital stay (days)	11 (5)	14 (6)	**0.052**	0.262	11 (5)	15 (6)	**0.050**	0.603
R0 resection			0.423	0.285			0.543	0.691
No	15 (65.2)	19 (52.8)			11 (50.0)	14 (63.6)		
Yes	8 (34.8)	17 (47.2)			11 (50.0)	8 (36.4)		
LN harvest	21 (10)	25 (13)	0.224	0.307	21 (11)	25 (12)	0.281	0.332
LN ratio	0.05 (0.09)	0.06 (0.09)	0.574	0.111	0.05 (0.09)	0.08 (0.10)	0.290	0.315
Total costs (euro)	16,651 (6928)	17,728 (5258)	0.520	0.181	16,729 (7081)	18,090 (5700)	0.487	0.212

Bold values are statistically significant. It allows to underline the important parameters

*LDP* laparoscopic distal pancreatectomy, *ODP* open distal pancreatectomy, *SMD *standardised mean difference, *C–D* Clavien–Dindo, *CR-POPF *clincally relevant post-operative pancreatic fistula, *PPH* post-pancreatectomy haemorrhage, *DGE* delayed gastric emptying, *LN* lymph node

*Not computable

## Discussion

By applying the PSM analysis, the present study demonstrated that LDP for patients with body-tail pancreatic neoplasms, including patients with PDAC, was comparable to traditional open procedures in terms of operative findings, postoperative results, oncological outcomes and total costs. This is the first single-center study which utilised PSM analysis to compare LDPs and ODPs plus splenectomy with regard to both benign and malignant body-tail pancreatic neoplasms. The previous study of Nakamura et al. [10] regarded only benign or low-grade malignant tumors, excluding patients with a preoperative diagnosis of pancreatic cancer and considered LDP and ODP with or without splenectomy. In addition, the other studies which utilised PSM analysis regarded only pNETs [14] or PDAC [11, 13]. The PSM analysis can be used in observational/retrospective cohort studies reducing selection bias by equating the groups compared. Thus, it allows reliable results and robust evidence, avoiding randomized controlled trials. Moreover, the two RCTs [8, 9] reported a small and heterogeneous sample. The LEOPARD study [8] analysed 108 patients of whom 51 had undergone minimally invasive distal pancreatectomy (MIDP) and 57 had undergone ODP. In addition, of the 51 assigned to MIDP, 4 were excluded; of the remaining 47, 42 underwent LDP and 5 robotic distal pancreatectomy. The second RCT [9] included only 58 patients, 29 who had undergone LDP and 29 ODP.

The usefulness of PSM analysis was clearly evident when comparing unmatched with matched populations (Table 1). Interestingly, in the unmatched populations, distinct differences in baseline patients and tumour characteristics between both modalities were present, and the criteria used to select patients for the laparoscopic approach were different with respect to those used to select patients for the open approach; the patients who underwent LDP were significantly younger and had fewer comorbidities than those who underwent ODP. In addition, the tumours of those undergoing LDP were significantly smaller, more frequently benign and were located in the tail of the pancreas with respect to those undergoing ODP. These data may be explained by the fact that the tumors located in the pancreatic body were closed to the major vessels. In addition, the current study included the very first LDPs in which the learning curve of the surgeons had not been completed. Subsequently, with increasing experience, the surgeons extended their indication for LDP to include tumors located in the pancreatic body, older patients and also a greater number of malignant tumours. These data widely justified the use of a PSM analysis to compare well-balanced cohorts of patients, and to obtain reliable results and robust evidence.

Therefore, by applying PSM to all the patients with body-tail pancreatic neoplasms, 75 patients in each arm (Table 1) were analysed. This sample was larger than the two RCTs previously published in the literature [8, 9] and allowed showing that length of hospital stay, blood loss, postoperative morbidity and total costs were significantly reduced in LDPs with respect to ODPs. All the other parameters (operative time, 90-day mortality, severe postoperative morbidity, CR-POPF, POPF grad C, PPH, DGE, reoperation, readmission) were similar between the two groups (Table 2).

The shorter duration of the length of hospital stay reinforced the data from the two RCTs [8, 9], the multicenter study of Nakamura et al. [10] and from the several meta-analyses previously published [2, 22, 23]. In their systematic review and meta-analysis, Pericleous et al. [2] reported a reduced hospital stay of 2.7 days in favour of LDP; in the first RCT which compared LDP and ODP, de Rooij et al. [8] reported a length of hospital stay of 2 days less in patients who underwent LDP with respect to those who underwent ODP. Bjornsson et al. [9] reported a reduced length of hospital stay of 1 day. Finally, Nakamura et al. [10] showed a hospital stay of 23 ± 18 and 18 ± 14 days, respectively, for patients who underwent ODP and LDP. In the present study, LDP compared with ODP was associated with a 2-day reduction in a hospital stay. It should be pointed out that all the patients who underwent LDP or ODP in the Institute in the present study were treated with the same enhanced recovery principles in the postoperative period. In particular, discharge with surgical drainage in situ was carried out in both LDP and ODP patients before drain amylase levels had normalised to a daily output of 20 cc. The shorter duration of the length of hospital stay seems to be related to the reduction of postoperative morbidity rate and, subsequently, to the lower total costs. The reduction in blood loss and postoperative complications was mainly due to the advantage of the laparoscopic technique. Magnification of the structure and vessels allowed the best haemostasis which seemed to explain the reduction in blood loss. In addition, it is well known that the reduction in blood loss [20] is related to a lower incidence of overall postoperative complications, even if severe complications were similar between LDP and ODP. These data indicated that the mini-invasive approach seemed to be useful in reducing minor complications as well as wound infections, and also that it was a safe approach for distal pancreatectomy. Finally, minimally invasive surgery typically increases the total costs due to the initial cost of the laparoscopic system. However, in the present study, the total cost of LDP was significantly lower than ODP. This fact was probably due to the reduced length of hospital stay with a reduced time to functional recovery and a lower postoperative complication rate. This confirmed the results of other studies [24, 25] regarding the benefit of LDP as a promising treatment option from a health economic perspective.

Applying PSM to the patients with body-tail PDAC, 22 patients in each arm (Table 3) were analysed. The present study was the fourth which utilised PSM analysis to compare LDP and ODP for left-sided PDAC. Chen et al. [12] reported 66 well-matched patients in each group and concluded that LDP was a safe, feasible and favourable approach in short-term surgical outcomes (less blood loss, shorter operative time and shorter hospital stay). In addition, patients who underwent LDP rather than ODP for PDAC had comparable oncological results. Raoof et al. [11] reported a retrospective case–control study of patients who underwent distal pancreatectomy for pancreatic adenocarcinoma, selected from the National Cancer Database. After PSM, two well-balanced groups of 563 patients each were analysed; the patients who underwent an LDP had oncological outcomes comparable to those of the patients who underwent ODP. A shorter duration of hospital stay was observed in the laparoscopic group. Finally, van Hilst et al. [13] stated that comparable results were seen after minimally invasive distal pancreatectomy (MIDP) and ODP for PDAC. In the present study, LDP had a shorter length of hospital stay and less postoperative morbidity than ODP; the oncological outcomes were comparable between the two approaches (Table 4) reinforcing the results of the previous studies. Currently, these four studies represent the highest level of evidence in the literature regarding the comparison between LDP and ODP in patients with PDAC. However, the study of Raoof et al. [11] was based on the National Cancer Database, the study of Chen et al. [12] and the present study regarded a single centre experience and, finally, the study of van Hilst et al. [13] represents a multicentre pan-european study. It should be noted that, in the present study, the results of comparing LDP and ODP in patients with PDAC are similar to those in patients with any type of body-tail pancreatic neoplasms. This was probably due to the fact that LDP for PDAC was performed when the surgeons’ learning curve had been completed, and the surgeons were appropriately trained. The current study seemed to suggest that LDP for patients with PDAC was not inferior to ODP in terms of operative findings, postoperative and oncological outcomes, and total costs. The ongoing DIPLOMA trial (ISRCTN44897265; www.e-mips.com) will additionally clarify the role of LDP in the setting of ductal adenocarcinoma of the pancreas.

The current study has some limitations: for the most part, the retrospective design, the small sample size, the small number of laparoscopic resected patients per years and a long period of observation in a single tertiary centre. However, it should be pointed out that to overcome selection bias in retrospective studies, the most effective method (PSM analysis) of obtaining well-balanced groups to compare was carried out, thus avoiding randomisation and assuring reliable results.

In conclusion, despite its limitations, the present study, by applying PSM analysis, seemed to confirm that LDP, for patients with body-tail pancreatic neoplasms, including PDAC, yielded comparable results in terms of operative findings, postoperative and oncological outcomes, and total costs with respect to ODP. In addition, LDP seemed to be superior to ODP regarding length of hospital stay, blood loss and overall postoperative complications. Total costs also seemed to be reduced when using the laparoscopic approach with respect to the open procedure. Finally, the present study seemed to suggest that LDP could be considered to be a standard approach in patients with both benign and malignant body-tail pancreatic lesions whereas open procedures should be indicated in selected cases.

## Data Availability

Data reported are available, included in our database of pancreatic cancer.

## Code availability

n.64/2017/U/Oss.
